# Novel *GFM2* variants associated with early-onset neurological presentations of mitochondrial disease and impaired expression of OXPHOS subunits

**DOI:** 10.1007/s10048-017-0526-4

**Published:** 2017-10-26

**Authors:** Ruth I. C. Glasgow, Kyle Thompson, Inês A. Barbosa, Langping He, Charlotte L. Alston, Charu Deshpande, Michael A. Simpson, Andrew A. M. Morris, Axel Neu, Ulrike Löbel, Julie Hall, Holger Prokisch, Tobias B. Haack, Maja Hempel, Robert McFarland, Robert W. Taylor

**Affiliations:** 1 Wellcome Centre for Mitochondrial Research, Institute of Neuroscience, The Medical School, Newcastle University, Newcastle upon Tyne, NE2 4HH UK; 2 Department of Medical and Molecular Genetics, King’s College London School of Medicine, London, UK; 3 Division of Evolution and Genomic Sciences, School of Biological Sciences, University of Manchester, Manchester, UK; 4 Alder Hey Children’s Hospital NHS Foundation Trust, Liverpool, UK; 5 University Children’s Hospital, University Medical Center Hamburg-Eppendorf, Hamburg, Germany; 6 Department of Diagnostic and Interventional Neuroradiology, University Medical Center Hamburg-Eppendorf, Hamburg, Germany; 7 Department of Neuroradiology, Royal Victoria Infirmary, Newcastle upon Tyne, UK; 8 Institute of Human Genetics, Helmholtz Zentrum München, Oberschleißheim, Germany; 9 Institute of Human Genetics, Technische Universität München, Munich, Germany; 10 Institute of Medical Genetics and Applied Genomics, University of Tübingen, Tübingen, Germany; 11 Institute of Human Genetics, University Medical Center Hamburg-Eppendorf, Hamburg, Germany

**Keywords:** WES, *GFM2*, Mitochondrial translation, Developmental delay, Mitochondrial disease

## Abstract

Mitochondrial diseases are characterised by clinical, molecular and functional heterogeneity, reflecting their bi-genomic control. The nuclear gene *GFM2* encodes mtEFG2, a protein with an essential role during the termination stage of mitochondrial translation. We present here two unrelated patients harbouring different and previously unreported compound heterozygous (c.569G>A, p.(Arg190Gln); c.636delA, p.(Glu213Argfs*3)) and homozygous (c.275A>C, p.(Tyr92Ser)) recessive variants in *GFM2* identified by whole exome sequencing (WES) together with histochemical and biochemical findings to support the diagnoses of pathological *GFM2* variants in each case. Both patients presented similarly in early childhood with global developmental delay, raised CSF lactate and abnormalities on cranial MRI. Sanger sequencing of familial samples confirmed the segregation of bi-allelic *GFM2* variants with disease, while investigations into steady-state mitochondrial protein levels revealed respiratory chain subunit defects and loss of mtEFG2 protein in muscle. These data demonstrate the effects of defective mtEFG2 function, caused by previously unreported variants, confirming pathogenicity and expanding the clinical phenotypes associated with *GFM2* variants.

## Introduction

Mitochondrial disorders are a genetically and clinically heterogeneous group of diseases which can arise due to defects in the oxidative phosphorylation (OXPHOS) system. The OXPHOS system comprises five multi-subunit protein complexes—the electron transport chain (complexes I–IV) and ATP synthase (complex V)—which function together to generate cellular energy in the form of ATP. Mitochondria possess their own circular, double-stranded 16,569 bp genome, encoding 22 transfer RNAs (tRNAs), 2 ribosomal RNAs (rRNAs) and 13 polypeptides [1]. All OXPHOS components, with the exception of complex II, are under bi-genomic control and as such are the product of both nuclear DNA and mitochondrial DNA (mtDNA)-encoded proteins [2]. Nuclear genes also encode the proteins responsible for mtDNA maintenance, mitochondrial transcription and translation and all other mitochondrial processes. As a consequence, mutations in either mitochondrial or nuclear genes may compromise ATP synthesis and cause mitochondrial disease. Extensive clinical and genetic heterogeneity makes the identification, characterisation and diagnosis of mitochondrial disease challenging, because clinical features often overlap with other neurological or systemic diseases. The advent of next-generation sequencing, specifically whole exome sequencing (WES), has improved the identification of disease-causing pathogenic variants in many different genes resulting in a much greater diagnostic yield than previous candidate gene screening approaches [3].

Mutations in genes involved in the translation of mtDNA are well-documented causes of mitochondrial disease [4, 5]. These genetic variants characteristically lead to combined OXPHOS deficiencies. The spectrum of resulting clinical phenotypes is broad, often manifesting as multi-system disease with the heart, skeletal muscle, brain and liver commonly affected [6]. The translational machinery within mitochondria is distinct to that of the cytosol and has several features which are reminiscent of the system employed in prokaryotic protein synthesis. This is reflective of the evolutionary origins of mitochondria as an endosymbiont α-proteobacterium [7]. Mitochondria possess their own mitoribosome which is a combination of 12S and 16S rRNAs, encoded within the mitochondrial genome, and at least 80 nuclear genes encode the proteins that form the large and small mitoribosomal subunits [8].

Pathogenic variants have been described in several of the mitoribosomal subunits [9–11]. Mitochondrial translation defects can also arise due to mutations in the mitochondrial tRNAs and in the mitochondrial protein translation factors [12]. Mitochondrial amino-acyl tRNA synthetases (mt-aaRSs) are a group of enzymes with an essential role within mitochondrial protein synthesis and are associated with a continuously expanding number of mitochondrial disease presentations. There are 19 mt-aaRSs, which utilise ATP to specifically catalyse the attachment of amino acids to their cognate tRNA [13]. Mitochondrial diseases resulting from mutations in genes encoding all 19 of the mt-aaRSs have now been described in the literature, with the most recently identified being *WARS2* [14].

The human genes *GFM1* and *GFM2* encode the proteins mtEFG1 and mtEFG2, respectively, both of which are homologs of the highly conserved bacterial translation elongation factor G (EF-G) [15]. EF-G functions at multiple stages within prokaryotic protein synthesis, with roles in the translocation of the ribosome during elongation and ribosome recycling upon termination or stalling of translation [16]. In human mitochondria, the dual roles of prokaryotic EF-G are carried out by the two distinct human homologs. Human mtEFG1 acts as an elongation factor with mitoribosome translocation activity, while mtEFG2 functions at the termination step of translation to disassemble the mitoribosome and allow subsequent cycles of mitochondrial protein synthesis [17].

Seventeen mitochondrial disease patients with *GFM1* mutations across 13 families have been described in the literature so far. Earlier cases were associated with very severe systemic disease with early or neonatal onset, resulting in death within the first 2 years of life. Some clinical features, such as microcephaly, liver disease and encephalopathy, were common to multiple cases. However, more recent publications have described a new, milder, disease progression associated with *GFM1* mutations, with survival at 6 and 7 years of age [18, 19].

Two families with gene defects in *GFM2*, identified through whole exome sequencing, have previously been described in the literature associated with Leigh syndrome, microcephaly, simplified brain gyral pattern and insulin-dependent diabetes [20, 21]. Here we present two unrelated patients with previously unreported variants in *GFM2*, documenting OXPHOS deficiencies in different tissues and expanding the clinical phenotypes associated with *GFM2*-related mitochondrial disease.

## Patients and methods

All studies were completed according to local Ethical Approval of the Institutional Review Boards of Newcastle University (the National Research Ethics Service Committee North East—Newcastle & North Tyneside 1) and of the Technische Universität München. In agreement with the Declaration of Helsinki, all individuals or their guardians provided written informed consent before undergoing evaluation and genetic testing.

### Patient 1

Patient 1, an 11-year-old male, is the first child of healthy non-consanguineous parents and has a healthy younger brother. Pregnancy was complicated by intrauterine growth restriction and he was born at term, in good condition, by normal vaginal delivery weighing 2.0 kg. He had asymptomatic hypoglycaemia in the neonatal period and mild jaundice and was tube fed initially. He subsequently had a urinary tract infection and required orchidopexy for an undescended right testis.

Developmental delay was first noted at 2.5 years in relation to language and communication skills; he had acquired his first words at 12–18 months, but did not put words together until aged 3 years. From 5 years onwards, he has become increasingly dysarthric. He has used a knife and fork from 3 years of age but has never been able to write. He started walking at 14 months and could run at 4 years but he subsequently developed a dystonic posture of his right foot and spasticity in both legs, leading to toe walking and loss of ambulation at 8 years; he currently mobilises by crawling. He has been continent since 2 years of age and continues to make slow academic progress. He has a normal head circumference, normal vision and hearing and no involuntary movements or seizures.

**Fig. 1:**
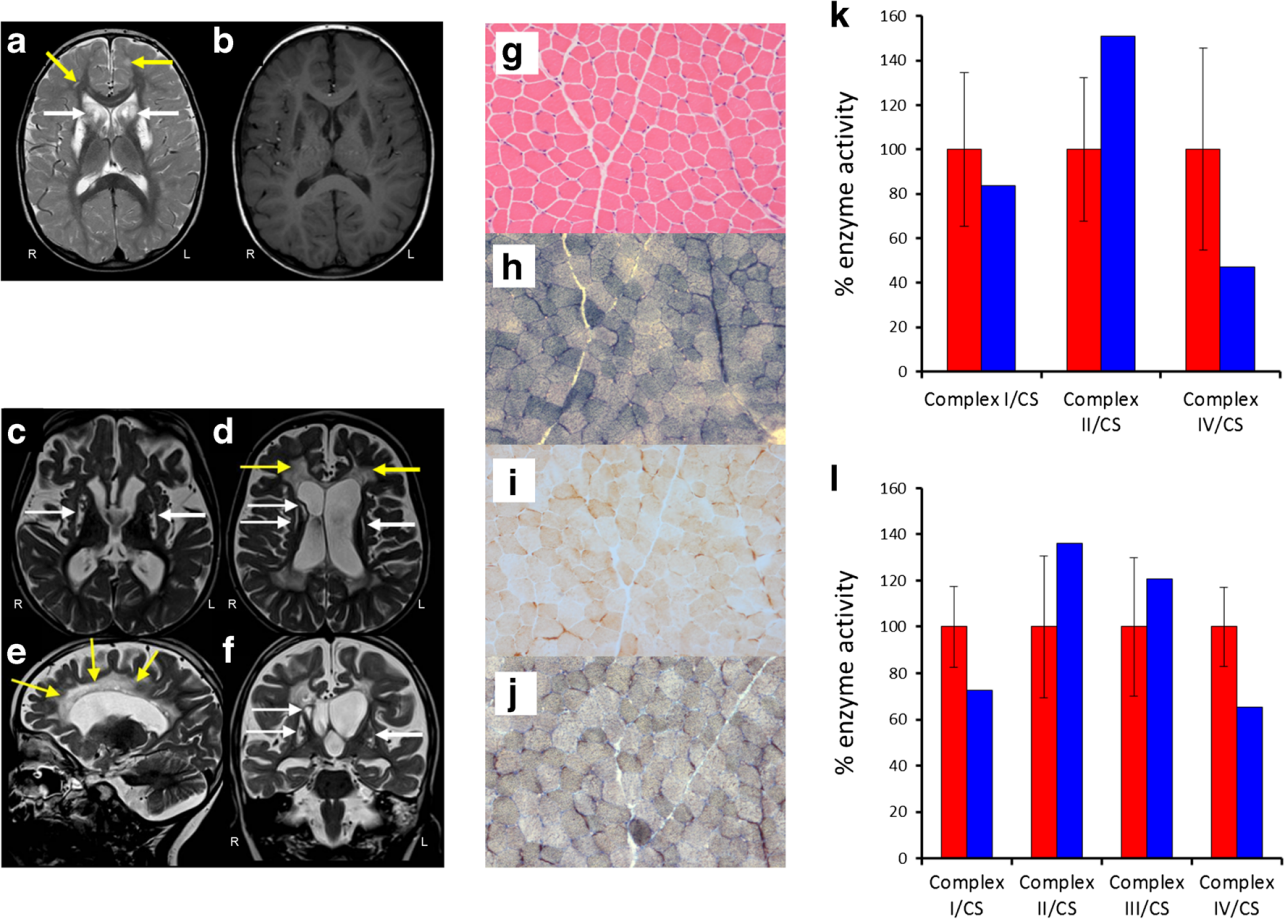
Cranial MRI, histochemical and biochemical investigations. MRI of Patient 1 showing bilateral T2 hyperintensities involving supratentorial white matter (yellow arrows), head of caudate nucleus (white arrows), putamen and genu and splenium of the corpus callosum (**a**) characterised by low T1 signal suggesting irreversible tissue damage (**b**). MRI of Patient 2 showing extensive T2 hyperintensities associated with volume loss involving bilateral periventricular and central white matter (**d**, **e**, yellow arrows) and defects involving both putamina and the head of caudate nucleus on the right (**c**, **d**, **f**, white arrows). Right (R) and left (L) are indicated. (**g**) Haematoxylin and eosin stain shows normal skeletal muscle morphology. Succinate dehydrogenase (SDH) (**h**), cytochrome *c* oxidase (COX) (**i**) and COX-SDH histochemistry (**j**) reveal a generalised and widespread COX deficiency. Respiratory chain enzyme activity measurements in skeletal muscle (**k**) and fibroblasts (**l**) demonstrate a severe complex IV defect in skeletal muscle and a mild complex I and IV defect in Patient 1 fibroblast cells compared to age-matched controls (red: controls, blue: patient 1)

Cranial MRI showed symmetrical bilateral high signal on T2-weighted images in the caudate, putamen and cerebellar dentate nucleus. There were also abnormalities in the corpus callosum and the subcortical white matter of the cerebral and, particularly, the cerebellar hemispheres, with further abnormal areas in the deep white matter (Fig. 1a, b). CSF lactate was elevated on two occasions at 3.2 and 3.4 mmol/L (normal range, 0.7–2.1 mmol/L). Sequencing of the mitochondrial genome and the *NFU1* gene were both normal.

### Patient 2

Patient 2, a 7-year-old female, is the second child of consanguineous parents originating from Syria. Born following an uneventful pregnancy at full term, development was unremarkable in the first 2 years of life. At the age of 2 years and 2 months, involuntary movements of the left hand were reported and within a few months, these had extended to involve all four limbs. Muscle strength and mass deteriorated and she lost the ability to walk at 4 years, to sit at 5 years and subsequently lost the ability to speak. At the age of 6 years, she presented with her first seizure and has subsequently developed a severe epilepsy disorder. Clinical assessment reveals severe global developmental delay, myopathic facies with an open mouth appearance and drooling, severe axial hypotonia with hypertonic limbs and dystonic involuntary movements. Communication was restricted to phonetic reading.

Lactate was repeatedly elevated in both serum (up to 4.1 mmol/L) and CSF (up to 3.1 mmol/L). Cranial MRI showed diffuse hyperintensities on T2-weighted imaging of the periventricular and central white matter with associated volume loss and atrophy of corpus callosum as well as T2 hyperintense defects of bilateral putamen and head of caudate nucleus (Fig. 1c–f). EEG revealed multifocal seizure activity.

### Histopathology and biochemistry

A diagnostic skeletal muscle biopsy was obtained from Patient 1 with informed consent; histological and histochemical investigations were carried out following standard procedures. The biochemical assessment of respiratory chain enzyme activities was undertaken as previously described in a skeletal muscle homogenate [22].

### Molecular genetic studies

WES was carried out for both patients to identify candidate pathogenic variants. Methodologies and variant filtering pipelines were carried out as previously described in [23, 24] for Patient 1 and Patient 2, respectively. Variants with a minor allele frequency (MAF) ≥ 0.01 (1%) on external sequencing databases such as the Genome Aggregation Database (gnomAD), and in-house control data sets, were excluded from the variant pool. Autosomal recessive variants (either compound heterozygous or homozygous) in genes known to encode proteins with mitochondrial function were then prioritised for further investigation.

Sanger sequencing of amplified gene products was carried out in accordance with BigDye® Terminator v3.1 Cycle Sequencing Kit manufacturing protocol. Sequencing primers for each variant were designed, using Primer3 (http://primer3.ut.ee), and sequences are available on request.

### Sequence analysis and in silico pathogenicity prediction

Sequence data was viewed and analysed using FinchTV. Variants are named according to transcript variant 1 of *GFM2* (GenBank accession number: NM_032380.4). Multiple sequence alignments were carried out using SeaView. Four freely available web-based programmes, Align GVGD (http://agvgd.iarc.fr/agvgd_input.php), SIFT (http://sift.jcvi.org), PolyPhen (http://genetics.bwh.harvard.edu/pph2/) and Combined Annotation Dependent Depletion (CADD) (http://cadd.gs.washington.edu/home), were used to predict the pathogenicity of the missense amino acid changes present in each patient through the analysis of Grantham differences and sequence homology.

### Cell culture

Primary fibroblast cell lines for both patients and paediatric controls were incubated at 37 °C in a humidified 5% CO_2_ atmosphere and grown in 75-cm^2^ flasks of 12 mL Gibco® MEM (minimum essential medium) containing 1 mM pyruvate, 2 mM l-glutamine and 4500 mg/L glucose, and supplemented with 10% FCS, 1× non-essential amino acids, 1× penicillin/streptomycin and 50 μg/mL uridine.

### Sample preparation, SDS-PAGE and Western blot analysis

Fibroblast cell pellets were re-suspended in lysate buffer (50 mM Tris/HCl pH 7.4, 130 mM NaCl, 2 mM MgCl_2_, 1 mM PMSF, 1% Nonidet P-40, 1× EDTA-free protease inhibitor), vortexed for 30 s and centrifuged at 1000*g* for 2 min, retaining the supernatant. Skeletal muscle (10-15 mg) was frozen in liquid nitrogen, then powdered and suspended in 1 mL RIPA buffer (1% IGEPAL, 0.5% sodium deoxycholate, 0.1% SDS, 1.5% Triton X-100, 10 mM beta mercaptoethanol, protease inhibitor (Pierce) and 1 mM PMSF in PBS). The muscle homogenate was then vortexed, incubated on ice for 45 min and subjected to 2 × 5-s homogenisations in a polytron tissue homogeniser. The final muscle lysates were prepared by centrifugation at 14,000*g* for 10 min at 4 °C, retaining the supernatant. Lysed fibroblast and muscle samples were incubated with dissociation buffer, containing 6.25 mM Tris/HCl pH 6.8, 2% SDS, 10% glycerol, 0.01% bromophenol blue and 100 mM DTT, for 20 min at 37° C.

All protein lysates were electrophoresed through 12% polyacrylamide resolving gels, cast, run and transferred on Bio-Rad Mini-Protean® Tetra Cell systems, at 200 V. Target proteins were interrogated by incubation overnight with antibodies at 4 °C and the resulting signals were visualised with ECL-prime (GE Healthcare) using Image Lab software on a BioRad Chemidoc MP. Antibodies used were the following: SDHA (ab14715; Abcam), ATP5A (ab110273; Abcam), ATP5B (ab14730; Abcam), UQCRC2 (ab14745; Abcam), MT-CYB (55090-1-AP; Proteintech Europe), MT-COI (ab14705; Abcam), MT-COII (ab110258; Abcam), NDUFB8 (ab110242; Abcam) and GFM2 (ab74874; Abcam).

### [^35^S] metabolic labelling

Assessment of de novo mitochondrial protein synthesis was carried out in patient and control fibroblasts grown to a confluency of 80%. Cells were washed three times for 10 min in methionine/cysteine-free media (Sigma, D0422) before adding 10% dialysed FCS (Sigma F0392) and 100 μg/mL emetine dihydrochloride to inhibit cytosolic translation. 20 μl  of [^35^S]-methionine/cysteine mix (Perkin-Elmer Easy Tag Express protein labelling mix, NEG-772, 73% L-met, 22% L-cys) was added to each flask and cells were incubated at 37 °C for 1 h. Cells were then washed in standard growth media containing methionine and harvested in PBS + 1 mM EDTA, pelleted at 200*g* for 4 min and resuspended in PBS + 1 mM PMSF and 1× EDTA-free protease inhibitor before being subjected to 15% SDS-PAGE as before. The gel was stained with Coomassie brilliant blue, fixed overnight (30% methanol, 10% acetic acid, 3% glycerol) and vacuum-dried prior to exposure using a PhosphorImage screen. Signal was detected using the Typhoon FLA9500 Phosphorimager and ImageQuant software (GE Healthcare) and mtDNA-encoded proteins were assigned to corresponding bands with reference to Chomyn et al. [25].

## Results

### Histopathological and biochemical studies

The muscle morphology of Patient 1 revealed no marked structural abnormalities visible upon staining with H&E (Fig. 1g). Oxidative enzyme histochemistry showed intense succinate dehydrogenase (SDH) activity (Fig. 1h) and a generalised decrease in cytochrome *c* oxidase (COX) activity across the biopsy (Fig. 1i). Sequential COX-SDH histochemistry confirmed widespread COX deficiency in the presence of normal SDH activity (Fig. 1j). The assessment of respiratory chain enzyme activities in muscle from Patient 1 revealed impairment of complex IV activity, while the activities of complexes I and II were both within normal limits (Fig. 1k). Interestingly, the fibroblast cells from this patient appeared to express a mild combined OXPHOS defect involving both complexes I and IV, albeit with residual enzyme activities of ~ 60% of normal (Fig. 1l).

### Variant identification, confirmation and segregation studies

**Fig. 2:**
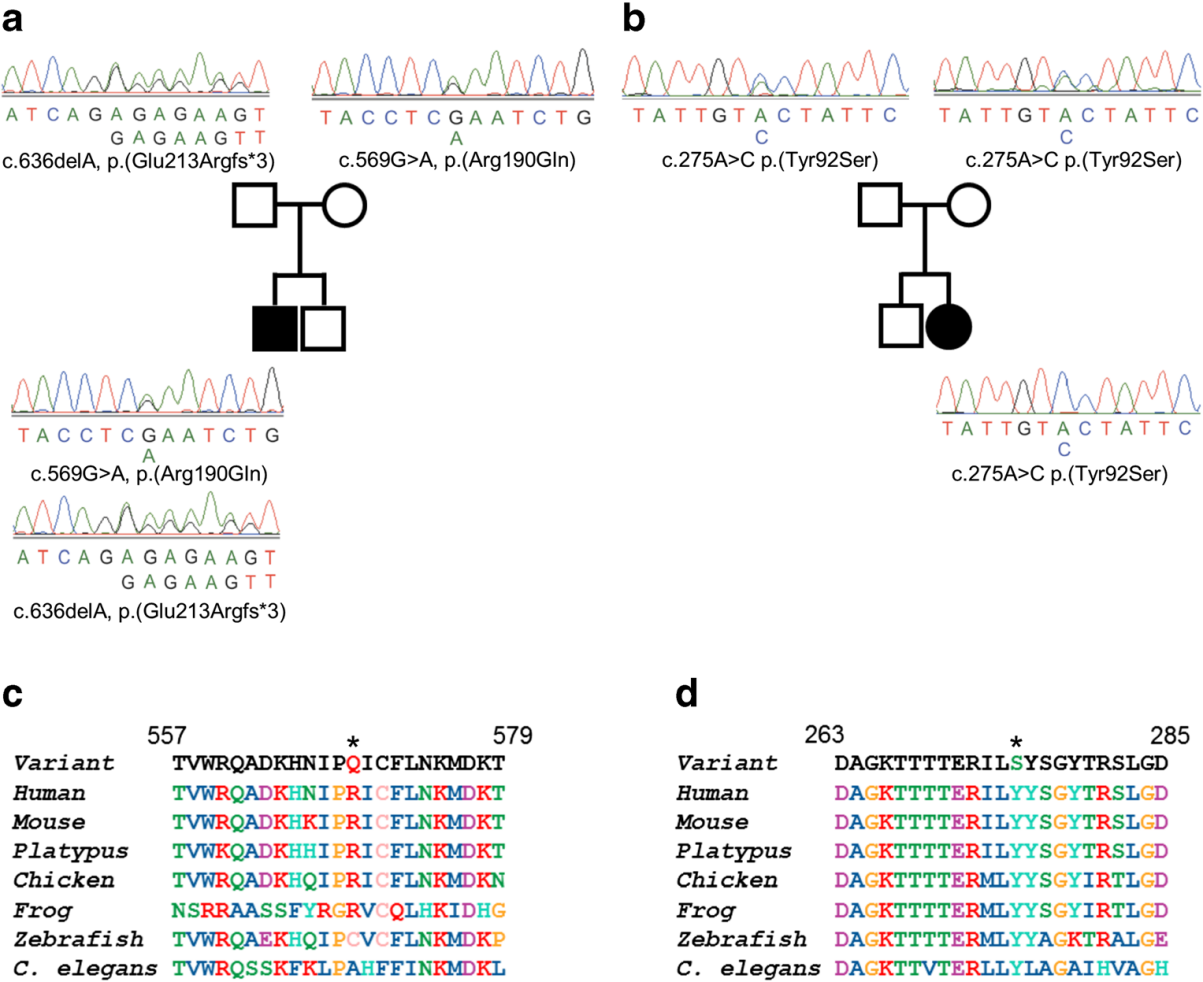
Segregation studies and missense residue conservation. **a** Familial pedigree and sequence data for Patient 1 and parents demonstrating recessive inheritance of compound heterozygous c.569G>A, p.(Arg190Gln) and c.636delA, p.(Glu213Argfs*3) *GFM2* variants. **b** Familial pedigree and sequence data for Patient 2 and parents demonstrating recessive inheritance of a homozygous c.275A>C, p.(Tyr92Ser) *GFM2* variant. Multiple sequence alignment of *GFM2* reveals moderate evolutionary conservation of the p.Arg190 residue (**c**) and the p.Tyr92 residue is invariant (**d**) (both residues shown by an asterisk)

WES of Patient 1 identified compound heterozygous variants in the *GFM2* gene, a c.569G>A single nucleotide substitution in exon 8 resulting in an arginine to glutamine missense change at residue 190 (p.(Arg190Gln)) and a c.636delA single nucleotide deletion in exon 9 producing a frameshift mutation and premature termination codon (p.(Glu213Argfs*3)) that can be classified as pathogenic without further work (Fig. 2a). WES of Patient 2 revealed a homozygous c.275A>C variant in exon 5 of *GFM2*, predicted to cause a tyrosine to serine missense change at residue 92 (p.(Tyr92Ser)) (Fig. 2b). Sanger sequencing of the probands plus their respective parents confirmed the segregation of the bi-allelic *GFM2* variants with disease in both families (Fig. 2a, b). All *GFM2* variants have been submitted to ClinVar (https://www.ncbi.nlm.nih.gov/clinvar/) with the following identifiers: c.569G>A, SCV000605919; c.636delA, SCV000605920; c.275A>C, SCV000605921.

### Missense residue conservation and in silico predictions of pathogenicity

Sequence conservation of each missense variant, along with the surrounding residues, showed that the p.Arg190 residue is conserved in mammals, but is less so in lower species. The region flanking the p.(Arg190Gln) change is moderately conserved (Fig. 2c). In contrast, the homozygous p.(Tyr92Ser) variant in Patient 2 involves a tyrosine that is conserved from humans through to yeast and the entire surrounding region is extremely well conserved (Fig. 2d).

All three *GFM2* variants are extremely rare; the c.564A>G, p.(Arg190Gln) allele, harboured by Patient 1, is present in 5/276,072 alleles on gnomAD (MAF = 0.00002), while the c.275A>C, p.(Tyr92Ser) variant present in Patient 2 in a homozygous state is entirely novel. In silico tools produced mixed predictions—the p.(Tyr92Ser) missense variant was classified as likely to affect protein function according to all four in silico prediction tools. SIFT and aGVFD classified the p.(Arg190Gln) change in Patient 1 as unlikely to interfere with protein function while PolyPhen was strongly supportive of a deleterious effect. CADD scores were 35 for c.564A>G and 28.7 for c.275A>C, predicting that these variants are in the top 0.01 and 0.1% of deleterious single nucleotide variants of the reference genome, respectively.

### Western blotting studies reveal varying OXPHOS defects

**Fig. 3:**
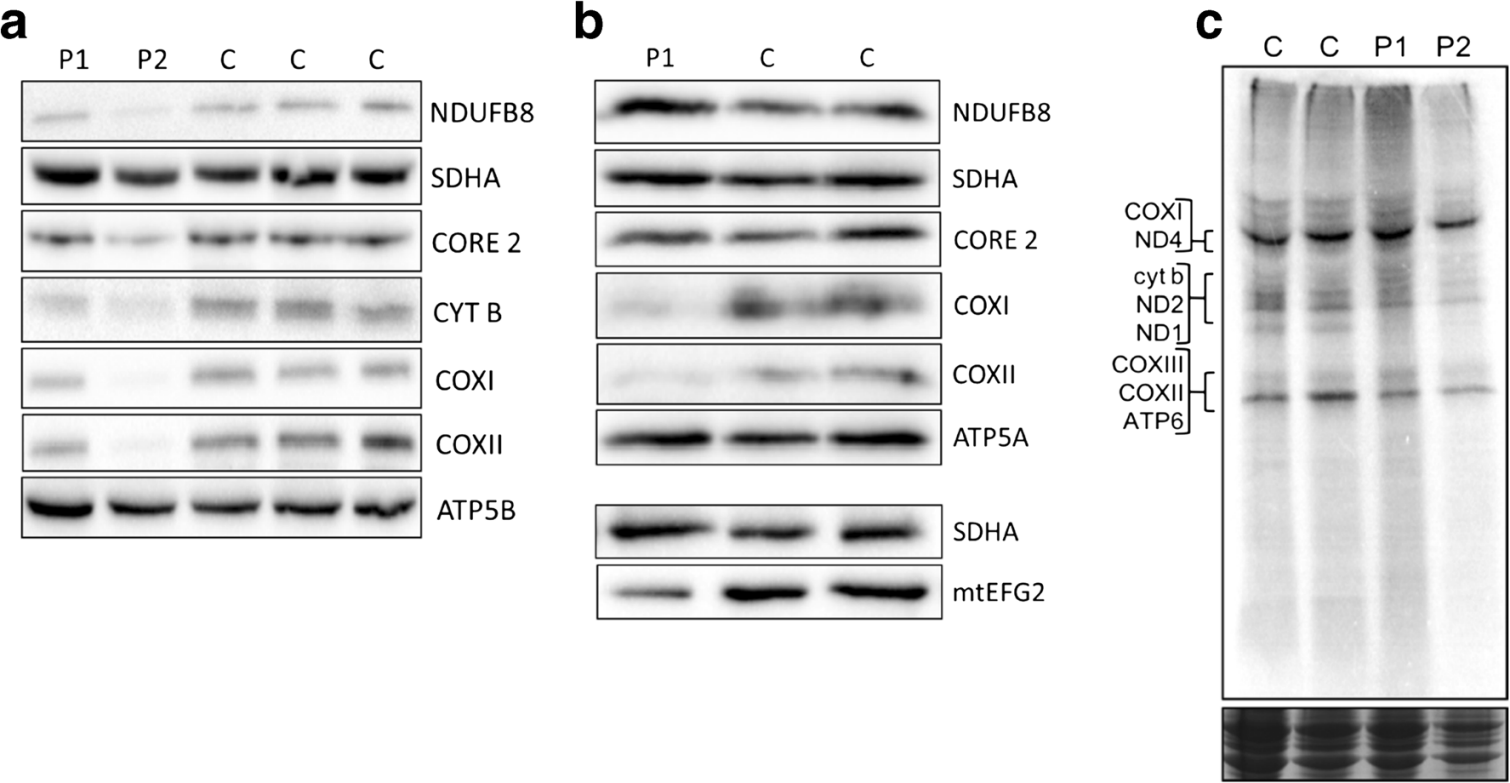
Western blot studies and a [^35^S] translational assay give insight into molecular effect of *GFM2* variants. **a** Fibroblast Western blot panel, with SDHA as a loading control. The panel demonstrates decreased steady-state levels of NDUFB8 (complex I), CORE 2 and CYT B (complex III) and COX I and COX II (complex IV) in the fibroblasts of Patient 2, but unchanged levels in the fibroblasts of Patient 1. **b** Skeletal muscle Western blot panel for Patient 1, with SDHA as a loading control. A complex IV deficiency is apparent, with decreased steady-state levels of COX I and COX II. Steady-state levels of other OXPHOS subunits remain unchanged. Levels of mtEFG2 protein are ~ 50% of controls. **c** [^35^S] methionine/cysteine incorporation in growing fibroblasts as a measure of de novo mitochondrial protein synthesis showed no difference between either Patient 1 or Patient 2 and the controls using Coomassie stain as loading control (bottom panel)

Western blot analysis was performed on the fibroblast lysates from both patients to investigate the effect of the *GFM2* variants on the steady-state levels of individual OXPHOS complex subunits. There was no apparent effect on the abundance of OXPHOS components in fibroblast cells from Patient 1 (Fig. 3a), consistent with the mild biochemical defects reported on direct enzyme assay. However, a clear decrease in steady-state levels of NDUFB8 (complex I), CYTB and CORE2 (complex III) and COXI and COXII (complex IV) was observed in the fibroblasts of Patient 2 compared to controls (Fig. 3a). In contrast to fibroblasts, skeletal muscle lysates from Patient 1 exhibited a marked decrease in steady-state levels of complex IV components, COXI and COXII (Fig. 3b). No skeletal muscle was available for Patient 2.

Investigation into the steady-state level of mtEFG2 was only possible following Western blot analysis of a skeletal muscle sample from Patient 1; fibroblast protein lysates repeatedly failed to produce a clear signal due to non-specificity of the commercial antibody in this tissue. The level of mtEFG2 protein in skeletal muscle from Patient 1 was approximately 52% of controls when quantified using Image Lab™ Software (Fig. 3b).

### [^35^S] translation assay

To assess whether the *GFM2* variants present in Patient 1 and Patient 2 cause any impairment of de novo mitochondrial protein synthesis in growing fibroblast cells, a [^35^S] metabolic labelling assay was performed. In the case of both Patient 1 and Patient 2, there does not appear to be any effect on the incorporation of [^35^S] methionine/cysteine into newly synthesised mitochondrial DNA-encoded proteins (Fig. 3c).

## Discussion

Through the application of whole exome sequencing, we have identified previously unpublished recessive *GFM2* variants in two unrelated patients with clinical features of mitochondrial disease and biochemical evidence of respiratory chain dysfunction. Western blot analysis of patient fibroblasts showed differential effects at the protein level for each of these patients. While Patient 2 shows decreased steady-state levels of mitochondrial-encoded OXPHOS subunits NDUFB8 (of complex I), COXI/COXII (of complex IV) and CYTB/CORE2 (of complex III), indicating a combined OXPHOS deficiency, steady-state OXPHOS protein levels were relatively unchanged in Patient 1 when compared to controls (Fig. 3a).

The gene *GFM2*, located at 5q13.3, encodes the protein mtEFG2 which has an essential role following the termination of mitochondrial translation as GTP hydrolysis is necessary for the recycling of the mitoribosome [17]. To date, *GFM2* variants have been identified in four patients (two sets of siblings), with phenotypes described as microcephaly, simplified gyral pattern and insulin-dependent diabetes in the first report and Leigh syndrome complicated by arthrogryposis multiplex congenita in the most recent family [20, 21]. While Leigh syndrome is a familiar mitochondrial phenotype, there are clinical aspects to these four cases that are rather atypical for mitochondrial disease, simplified gyral pattern and arthrogryposis multiplex. In contrast, the two cases reported here have a consistent clinical phenotype that is entirely compatible with mitochondrial disease and includes ‘red flag’ clinical features such as neurodevelopmental regression.

The steady-state levels of COXI and COXII in the skeletal muscle of Patient 1 were decreased (Fig. 3b), demonstrating a complex IV deficiency. These results are in accordance with the original diagnostic assessment of individual respiratory chain enzyme activities in Patient 1, which detected a complex IV activity of around 40% of controls. The residual levels of mtEFG2 in skeletal muscle from Patient 1 were approximately 50% of the two controls (Fig. 3b). The c.636delA p.(Glu213Argfs*3) *GFM2* mutation in Patient 1 causes a frame shift and a premature stop codon and is, therefore, likely to encode a non-functional truncated version of mtEFG2 that is likely to undergo nonsense-mediated mRNA decay. The 50% residual level of steady-state mtEFG2 seen in the skeletal muscle of Patient 1 can be explained by the p.(Arg190Gln) variant occurring in a region of the gene with only moderate sequence conservation. This is supported by the in silico pathogenicity predictions suggesting the missense change would not have major impacts on protein stability, consistent with the loss of only 48% normal mtEFG2 levels.

Steady-state OXPHOS subunit deficiency in Patient 1 is observable in skeletal muscle, but not fibroblasts, indicating tissue-specific differences. The deficiency of complex IV activity was most severe in the muscle (Fig. 1k) and less so in fibroblasts (Fig. 1l). This tissue-specific discrepancy in severity of OXPHOS defect is not uncommon in defects of mitochondrial translation and can arise due to the differences in ATP reliance in either tissue or differential levels of gene expression in the two tissue types. A number of deficiencies due to mt-aaRS mutations, such as *YARS2* encoding the mitochondrial tyrosyl-tRNA synthetase, show a much clearer OXPHOS defect in myoblasts than fibroblasts. In the case of pathogenic *YARS2* variants, a marked decrease in steady-state levels of NDUFB8 was observed only in myoblasts [26]. Muscle relies much more heavily on the generation of ATP through oxidative phosphorylation, and so variants in mitochondrial disease genes can on occasion present a much clearer effect at the steady-state protein level [27].

The clear difference in severity of OXPHOS defect between Patient 1 and Patient 2, visible upon Western blotting, can be explained by considering both the level of sequence conservation at the missense variant sites and in silico predictions of pathogenicity. There is consensus across two out of four prediction programmes (SIFT and aGVGD) that the p.(Arg190Gln) missense variant present in Patient 1 has a milder consequence compared to the p.(Tyr92Ser) *GFM2* variant of Patient 2. Despite Patient 1 having degradation of mtEFG2 from the allele containing the p.(Glu213Argfs*3) frameshift mutation, if the p.(Arg190Gln) amino acid change is less damaging and the remaining mtEFG2 protein retains some function, then it might be expected that the OXPHOS phenotype is less severe.

Despite mtEFG2 playing an important role in the termination step of mitochondrial translation, our [^35^S] translational assay showed no marked decrease in [^35^S] methionine/cysteine incorporation in growing fibroblasts as a measure of de novo mitochondrial protein synthesis, which may have been expected for Patient 1, due to a lack of clear phenotype in fibroblast cells. A possible explanation for the signal for Patient 2 being similar to controls can be found when the stage of translation at which mtEFG2 exerts its function is considered. In the majority of cases, mitoribosome recycling occurs upon completion of synthesis of a nascent peptide [28]. If mtEFG2 is dysfunctional, it will not prevent the incorporation of amino acids throughout the synthesis of a peptide, but will prevent the proper release of this product. Therefore, the signal coming from incorporated methionine/cysteine will not differ between control and patient cells.

In summary, we present two patients with developmental delay, dystonia, dysarthria and neuroimaging abnormalities in the putamen and caudate nuclei, along with subcortical white matter involvement who harbour previously unreported *GFM2* variants, identified through the application of WES. The histochemical, biochemical and genetic investigations, together with the functional data presented in this report, form compelling evidence that the *GFM2* variants identified through WES are pathogenic and causative of mitochondrial disease in both patients.
